# The complex genomic diversity of *Yersinia pestis* on the long‐term plague foci in Qinghai–Tibet plateau

**DOI:** 10.1002/ece3.10387

**Published:** 2023-07-28

**Authors:** Junrong Liang, Ran Duan, Shuai Qin, Dongyue Lv, Zhaokai He, Haoran Zhang, Qun Duan, Jinxiao Xi, Hua Chun, Guoming Fu, Xiaojin Zheng, Deming Tang, Weiwei Wu, Haonan Han, Huaiqi Jing, Xin Wang

**Affiliations:** ^1^ State Key Laboratory of Infectious Disease Prevention and Control, National Institute for Communicable Disease Control and Prevention Chinese Center for Disease Control and Prevention Beijing China; ^2^ Gansu Provincial Center for Disease Control and Prevention Lanzhou China; ^3^ Subei Mongolian Autonomous County Center for Disease Control and Prevention Jiuquan China; ^4^ Akesai Kazakh Autonomous County Center for Disease Control and Prevention Jiuquan China

**Keywords:** phylogenetic analysis, plague natural foci, Qinghai–Tibet plateau, SNP typing

## Abstract

Plague is a typical natural focus disease that circulates in different ecology of vectors and reservoir hosts. We conducted genomic population and phylogenetic analyses of the *Yersinia pestis* collected from the 12 natural plague foci in China with more than 20 kinds of hosts and vectors. Different ecological landscapes with specific hosts, vectors, and habitat which shape various niches for *Y. pestis*. The phylogeographic diversity of *Y. pestis* in different kinds plague foci in China showed host niches adaptation. Most natural plague foci strains are region‐and focus‐specific, with one predominant subpopulation; but the isolates from the Qinghai–Tibet plateau harbor a higher genetic diversity than other foci. The *Y. pestis* from *Marmota himalayana* plague foci are defined as the ancestors of different populations at the root of the evolutionary tree, suggesting several different evolutionary paths to other foci. It has the largest pan‐genome and widest SNP distances with most accessory genes enriched in mobilome functions (prophages, transposons). Geological barriers play an important role in the maintenance of local *Y. pestis* species and block the introduction of non‐native strains. This study provides new insights into the control of plague outbreaks and epidemics, deepened the understanding of the evolutionary history of MHPF (*M. himalayana* plague focus) in China. The population structure and identify clades among different natural foci of China renewed the space cognition of the plague.

## Graphical abstract

We conducted genomic population and phylogenetic analyses of the *Yersinia pestis* collected from 12 natural plague foci in China with more than 20 kinds of hosts and vectors. The evolutionary history and correlation between the genetic groups and plague foci of this study provide new insights into the control of plague outbreaks and epidemics, deepened the understanding of the evolutionary history of plague in China.

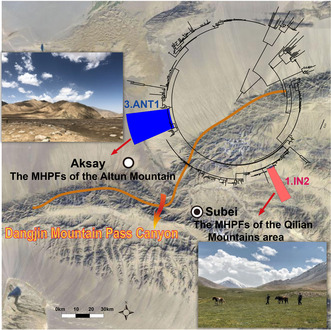

## 1 INTRODUCTION

*Yersinia pestis* is a highly virulent bacterium that diverged from *Y. pseudotuberculosis* around 5000–7000 years ago (Achtman et al., 1999, 2004). Plague is a rodent‐borne disease caused by this bacterium that has historically shown an outstanding ability to colonize and persist across different species, habitats, and environments (Meerburg et al., 2009; Rabiee et al., 2018). There have been three major outbreaks in human history, affecting a wide distribution including Asia, Eurasia, Africa, and American region. The first pandemic—the Justinian's plague, which occurred approximately between AD 541 and 767, spread in a continuous wave starting from the Mediterranean basin; its effects on humans, societies, and economies are still debated. The second pandemic wave was caused by *Y. pestis* biovar Medievalis, known as the “Black Death”. It started in 1346 and swept across Europe until the 19th century, resulting in the death of 30%–50% of the population. The third pandemic, caused by *Y. pestis* biovar Orientalis, started in Yunnan Province, China, reached Hong Kong in 1994, and spread around the world by steamboat and rail, lasting until the mid‐20th century. However, it did not cause as much damage as the previous two epidemics due to effective control (Bos et al., 2016; Khan, 2004; Perry & Fetherston, 1997; Zietz & Dunkelberg, 2004).

Plague has a long epidemic history in China. The third pandemic starting in western China and established sustained epizootic cycles worldwide, even in areas previously free of plague (Lynteris, 2017). Plague territories tend to be classified into primary reservoirs, vectors, landscapes, and genotypes of *Y*. *pestis*. There are 12 distinct natural plague foci throughout mainland China, found in 19 of the 31 provinces and autonomous regions (Ben‐Ari et al., 2012; Fang et al., 2011). Two of them documented on the Qinghai–Tibet plateau are *Marmota himalayana* plague focus (MHPF, detected in 1954) and *Microtus foscus* plague natural focus (MFPF, found in 1997) (Zhou et al., 2004). The former with biovar antiqua *Y*. *pestis* pathogens, the latter was biovar microtus. Before the 1990s, most human cases of plague were due to MHPF; since the 1990s, plague epidemics have erupted in southern China (mainly in Yunnan) (Wang et al., 2017); and since 2004, MHPF has reemerged and has been continually active, with outbreaks emerging year after year in conjunction with regional outbreaks, occasionally affecting human beings (Cui et al., 2013; He et al., 2021). Analysis of the spatiotemporal evolution and phylogenetic diversity of *Y*. *pestis* circulating in China is important for more effective control of its outbreaks and epidemics. At present, the local microevolution and phylogeography of *Y*. *pestis* among different natural plague foci in China remain unclear. Thus, we combined ecologic and genomic epidemiology to gain insights into the geographic epidemic and local microevolution of plague.

## 2 MATERIALS AND METHODS

### 2.1 *Yersinia pestis* isolate dataset

Eighty‐two strains collected from two main hosts: *M. himalayana* and *Microtus* were selected from the MHPF to clarify the evolutionary relationship and phylogenetic status of Qinghai–Tibet plateau plague foci of China. In order to define the global population structure and identify clades among different natural foci of China, we obtained a total of 544 *Y. pestis* with completed genomes or draft genomes from the NCBI database. Altogether, 381 *Y. pestis genomes* isolated from 14 provinces in China from 1940 to 2021; in addition, 245 *Y. pestis* strains published by other countries.

DNA from 82 *Y. pestis* was extracted using the Wizard® Genomic DNA Purification Kit (Promega) according to the manufacturer's instructions (Miller et al., 1988). Library preparation was performed using the Nextera XT Library Prep Kit (Illumina). The libraries were sequenced on an Illumina platform with a minimum of 100‐fold coverage.

### 2.2 Availability of data

The whole genome used in this study and phylogenetic analysis information are available in Table S1.

### 2.3 SNP calling and phylogenetic analysis

The quality of the raw paired‐end reads were evaluated using FastQC (v0.11.8) with the parameters: fastqc ‐q read_R1.fastq.gz ‐o outputDir (de Sena & Smith, 2019). We did quality filter with fastp (version 0.20.1) to provide clean data for downstream analysis: fastp ‐i in.R1.fq.gz ‐I in.R2.fq.gz ‐o out.R1.fq.gz ‐O out.R2.fq.gz. The filtered short reads were assembled using the SPAdes 3.15.4 with the parameters: spades.py ‐t 40 ‐1R1.clean.fq.gz ‐2R2.clean.fq.gz ‐o output (Prjibelski et al., 2020). To constructed the phylogenetic relationships among the 626 isolates, the genome alignment of strains were computed by Snippy v4.4.5 (Bush, 2021). SNPs between the contigs and reference genome *Y. pestis* CO92 (NC_003143.1) were found with the default parameters: snippy ‐cpus 16 ‐outdir mysnps ‐ref CO92.gbk ‐R1 R1.fastq.gz ‐R2 R2.fastq.gz (https://github.com/tseemann/snippy). Recombinant regions of the full SNP alignment were identified using the software package Gubbins v3.0.0 (Croucher et al., 2015) to exclude the SNPs in recombinant regions for the following analysis. Trees were visualized using either FigTree v.1.4.4 (https://github.com/rambaut/figtree) or iTol (Letunic & Bork, 2021). We compared the SNP characteristics of *Y. pestis* from multiple hosts among different plague foci in China and created a pairwise SNP distance matrix to understand the host–vector–pathogen interactions in association with changes in the genome profile. The packages of ggplot2 (Ito & Murphy, 2013) in R (v3.6.1) were used to draw frequency distribution histograms of SNP distances between samples and intergroup box plots.

### 2.4 Bayesian population structure analyses

Bayesian analysis with the program STRUCTURE v2.3.4 was used to assign the number of potential clusters present in the SNPs of all the 626 *Y. pestis* with a Markov Chain Monte Carlo (MCMC) algorithm (Wagner & Zhang, 2011). STRUCTURE was run with a burn‐in period of 10,000 and MCMC simulation of 30,000 iterations. The ancestry model was the “admixture model,” and the frequency model was “allele frequency correlated”. In addition, runs for each cluster (*K*) = 1–10 were replicated four times. To determine the number of clusters to show how the isolates could be grouped, delta *K* was calculated using STRUCTURE Harvester Ver. 0.6.93 software (Evanno et al., 2005).

### 2.5 Pan‐genome construction and function analysis of unique genes

To compare of the genetic traits among isolates from the 12 natural foci of China, all 381 genomes of *Y. pestis* from China were used for pan‐genome analysis. The genome sequences were annotated using the Prokka v1.14.5 (Seemann, 2014). We obtained the clustering of all protein sequences, and then characterized the core, accessory, and unique genes with CD‐HIT to gain insight into the features of the pan‐genome (Fu et al., 2012). The core genome included shared genes among all of the genomes, while the accessory genome contained genes shared by at least two strains; the remaining genes in only one strain were unique genes. The reads mapping were did to align the reads to the unique genome with Bowtie (Langmead & Salzberg, 2012). According to the sequence coverage of the unique genes, we can exclude the possibility of the contaminant. To better understand the functional features of the unique genes, the COG database was used for functional classification. All unique genes were searched against the COG database using BLASTp with an *E* value of 1e‐5 (Tatusov et al., 2000).

## 3 RESULTS

### 3.1 Genomic population structure of *Yersinia pestis*

**FIGURE 1:**
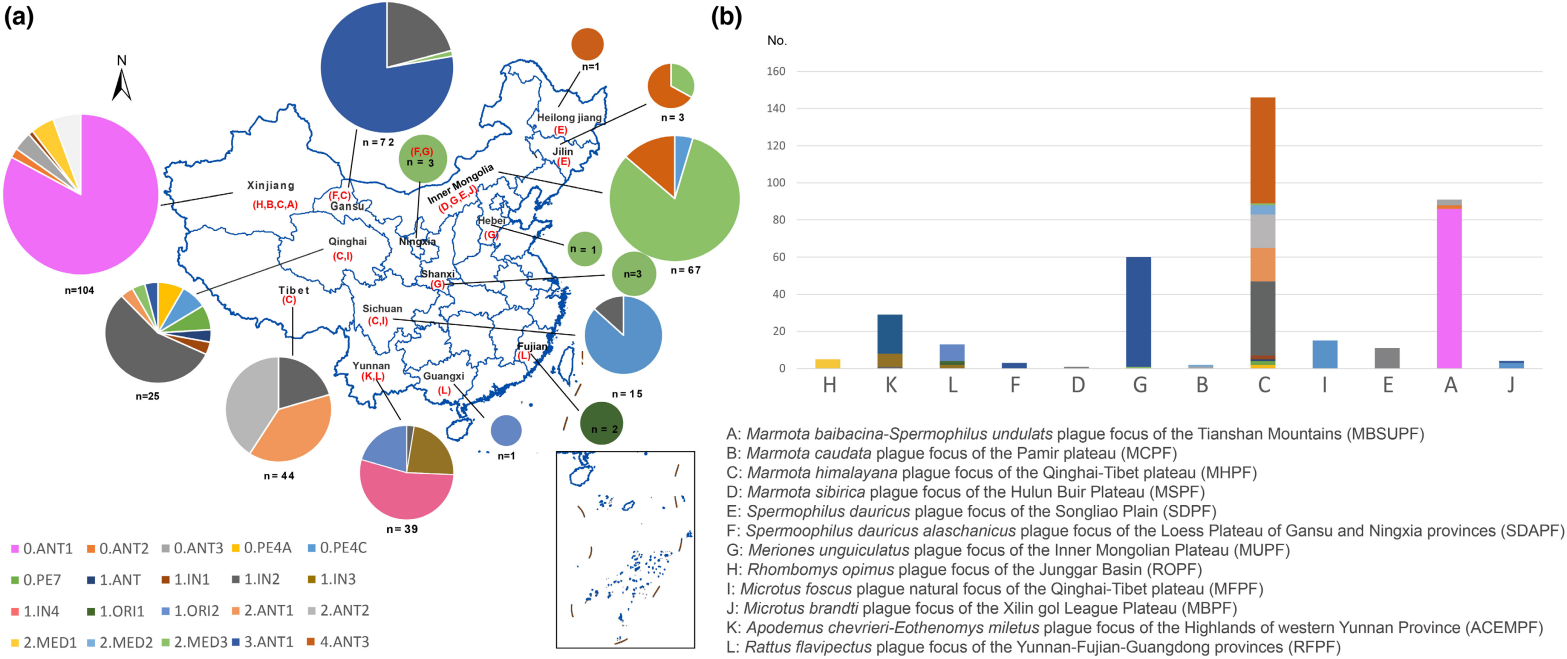
The genomic population and spatial distribution of *Yersinia pestis* isolates. (a) Distribution of genomic population of *Y. pestis* recovered from different provinces of China. (b) Distribution of genomic population of *Y. pestis* recovered from different natural foci. The 12 natural foci are shown in capital letters.

**FIGURE 2:**
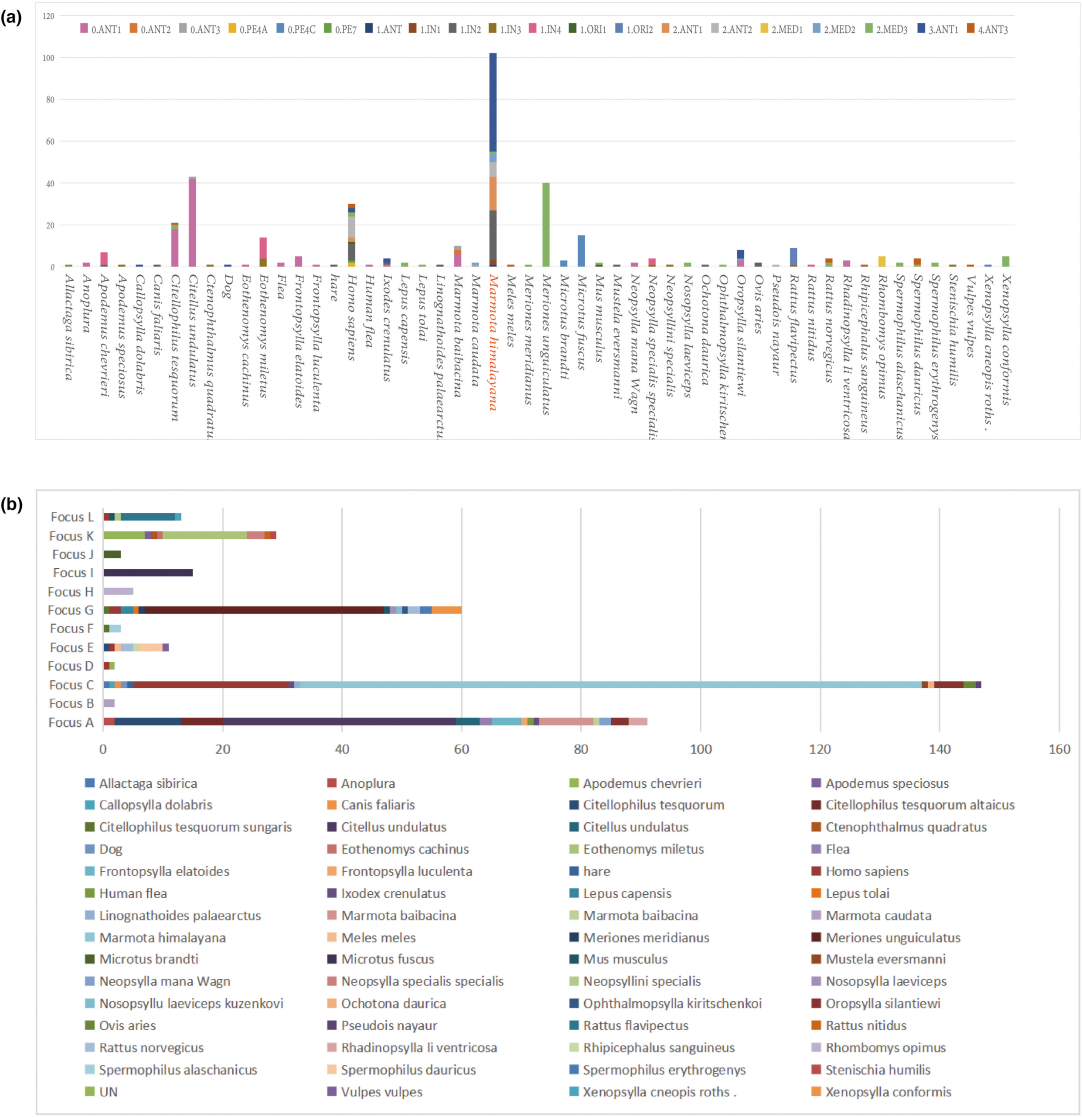
(a) Distribution of genomic subpopulation of *Yersinia pestis* recovered from different host populations. (b) The diversity of hosts in each of the foci. Focus A: *Marmota baibacina–Spermophilus undulats* plague focus of the Tianshan Mountains (MBSUPF). Focus B: *Marmota caudata* plague focus of the Pamir Plateau (MCPF). Focus C: *Marmota himalayana* plague focus of the Qinghai–Tibet Plateau (MHPF). Focus D: *Marmota sibirica* plague focus of the Hulun Buir Plateau (MSPF). Focus E: *Spermophilus dauricus* plague focus of the Songliao Plain (SDPF). Focus F: *Spermoophilus dauricus alaschanicus* plague focus of the Loess Plateau of Gansu and Ningxia provinces (SDAPF). Focus G: *Meriones unguiculatus* plague focus of the Inner Mongolian Plateau (MUPF). Focus H: *Rhombomys opimus* plague focus of the Junggar Basin (ROPF). Focus I: *Microtus foscus* plague natural focus of the Qinghai–Tibet Plateau (MFPF). Focus J: *Microtus brandti* plague focus of the Xilin gol League Plateau (MBPF). Focus K: *Apodemus chevrieri–Eothenomys miletus* plague focus of the Highlands of western Yunnan Province (ACEMPF). Focus L: *Rattus flavipectus plague* focus of the Yunnan‐Fujian‐Guangdong provinces (RFPF).

The 626 *Y. pestis* genomes represented isolates from 28 countries between 1905 and 2021 (Table S1). The majority originated from China (60.9%), Russia (16.6%), the United States (14.4%), and Georgia (1.8%). Altogether, 381 *Y. pestis* genomes were isolated from 14 provinces in China, including Tibet, Qinghai, Xinjiang, Gansu, Inner Mongolia, Heilongjiang, Jilin, Ningxia, Shaanxi, Yunnan, Fujian, Guangxi, Hebei, and Sichuan (Figure 1a). Each of the 12 natural foci of China included more than one strain, with *Y*. *pestis* isolated from diverse sources (Figures 1b and 2a). There distributed the most diversity types of genomic subpopulation of *Y. pestis* from *M. himalayana* and Homo sapiens (Figure 2a). The highest diversity of hosts was detected in MHPF, with 14 kinds of host (Figure 2b).

**FIGURE 3:**
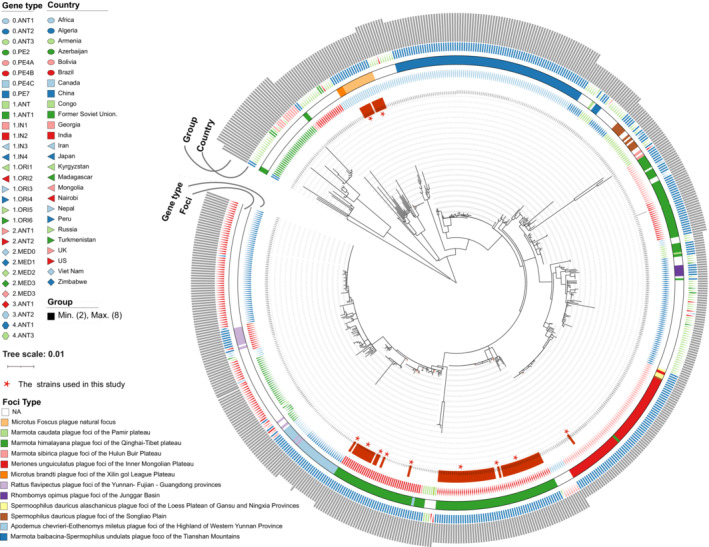
The maximum‐likelihood phylogenetic tree of 626 *Yersinia pestis* genomes. The phylogenetic tree was constructed based on mutational SNPs across the whole core genome. The tree is colored based on the isolation regions, branch lineages, and natural foci. The strains sequenced in this study were marked in red stars.

We identified 1800 core‐genome SNPs among the 626 genomes after removing recombinant regions. Then a recombination‐filtered core‐genome maximum likelihood phylogeny was constructed based on these SNPs with *Y. pseudotuberculosis* IP 32953 as an outgroup. Two Antiqua strains belonging to the MHPF formed the deepest population 0.PE7, first emerging from the root of the tree before the first pandemic. This was followed by the human nonpathogenic stains of the *Microtus brandti* plague foci of the Xilin gol League focus (MBPF) and the MFPF. This suggests that the strains from these two *M. foscus* plague natural foci are ancient and distinct from other ancient typical strains (Figure 3).

### 3.2 High diversity of *Yersinia pestis* strains isolated from MHPF

Most of the *Y. pestis* isolated from MHPF were at the root of the evolutionary tree and scattered over many of the phylogenetic branches. Isolates from the Russia, Former Soviet Union and Georgia belong to population 0.PE2, which evolved more recently from the shared common ancestor with 0.PE7 of MHPF. The 0.PE4A of MHPF rooted of the 0.PE4, and with other subpopulations (0.PE4B and 0.PE4C) evolved from a common ancestral. The 2MED.1 of Russia and strains from *Rhombomys opimus* plague focus of the Junggar Basin shared the same ancestors of lineage 2MED.2 (MHPF). Isolates in population 3.ANT1 restricted to MHPF represent a distinct branch (Figure 3).

The average phylogenetic diversity among isolates within MHPF is greater than other foci. The 147 strains from MHPF were scattered into 11 lineages. Altogether, 3.ANT1 predominant in MHPF with 38.8%; 1.IN2 with 27.2%, 2.ANT2 and 2.ANT1 with 12.2%, respectively (Figure 1b). Although both located in the MHPF, the *Y. pestis* in the Altun Mountains and the Qilian Mountains in Gansu province showed two entirely different populations: 3.ANT1 was predominantly 98.25% (*n* = 56) in the Altun Mountains, but the Qilian Mountains entirely with 1.IN2 (*n* = 14). Our data suggested that a geographic landform served as a natural barrier to the movement and mixing of rodents, leading to a sequestration of their associated *Y. pestis* subpopulations within these regions.

### 3.3 Biogeographical distribution of strains from other foci

The genome sequences of other foci were much conserved with one main cluster. Isolates in *R. opimus* plague focus of the Junggar Basin with 100% of 2.MED1; isolates in *Spermoophilus dauricus alaschanicus* plague focus of the Loess Plateau of Gansu and Ningxia provinces with 100% of 2.MED3; MFPF with 100% of 0.PE4C; *Marmota sibirica* plague focus of the Hulun Buir Plateau and *S. dauricus* plague foci of the Songliao Plain with 100% of 4.ANT3; *Meriones unguiculatus* plague focus of the Inner Mongolian Plateau with 100% of 2.MED3, MBSUPF with 94.5% of 0.ANT1, MBPF with 100% of 0.PE4C, *Apodemus chevrieri–Eothenomys miletus* plague focus of the Highlands of western Yunnan Province with 70% of 1.IN4, *Rattus flavipectus* plague focus of the Yunnan‐Fujian‐Guangdong provinces with 64.29% of 1.ORI2 (Figure 1b).

Phylogenetic analysis indicated that the 1.IN4 isolates in western China (the wild rat: *A. chevrieri–E. miletus* plague focus of the Highlands of western Yunnan Province) originated from the 1.IN2 of MHPF (Figure 3, Figure S3). Then plague may have been introduced from the wild rat to the house mouse in western China (*R. flavipectus* plague focus of the Yunnan‐Fujian‐Guangdong provinces) with the present of distinct lineages 1.ORI2 (Figure 3, Figure S3). This is consistent with the current view that the third plague pandemic initially spread from the *M. himalayana* plague foci of the Qinghai–Tibet plateau to Yunnan.

### 3.4 Bayesian population structure analysis

**FIGURE 4:**
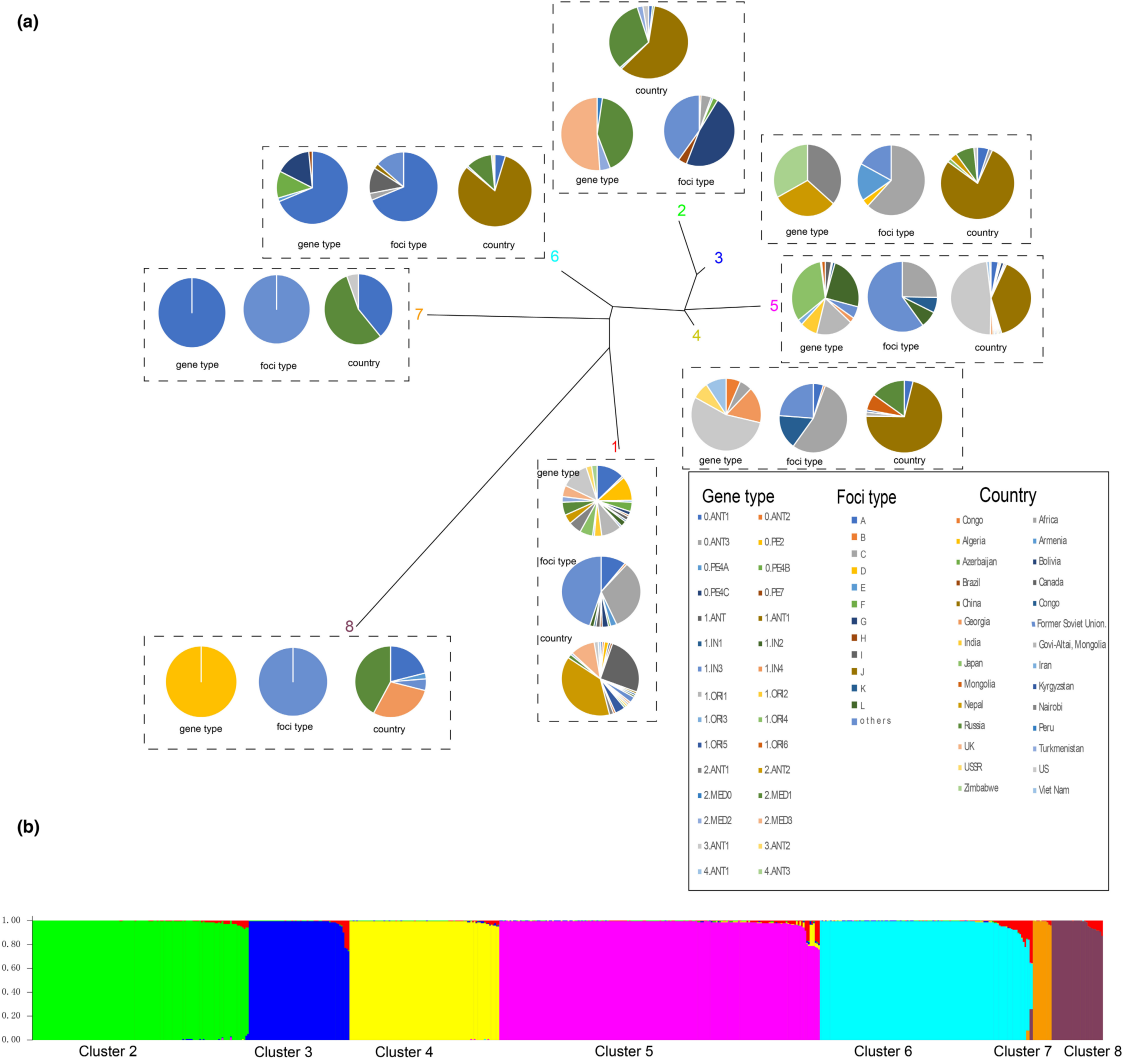
Genetic structure of the *Yersinia pestis*. (a) The country, focus type, and branch lineages included in each genetic group. (b) Graphical Bayesian cluster analysis using STRUCTURE, with the dataset for the most likely *K* (*K* = 8). Each color corresponds to a suggested cluster. High admixture between clusters was observed in Group 1, which is shown in red. Focus A: *Marmota baibacina–Spermophilus undulats* plague focus of the Tianshan Mountains (MBSUPF). Focus B: *Marmota caudata* plague focus of the Pamir Plateau (MCPF). Focus C: *Marmota himalayana* plague focus of the Qinghai–Tibet Plateau (MHPF). Focus D: *Marmota sibirica* plague focus of the Hulun Buir Plateau (MSPF). Focus E: *Spermophilus dauricus* plague focus of the Songliao Plain (SDPF). Focus F: *Spermoophilus dauricus alaschanicus* plague focus of the Loess Plateau of Gansu and Ningxia provinces (SDAPF). Focus G: *Meriones unguiculatus* plague focus of the Inner Mongolian Plateau (MUPF). Focus H: *Rhombomys opimus* plague focus of the Junggar Basin (ROPF). Focus I: *Microtus foscus* plague natural focus of the Qinghai–Tibet plateau (MFPF). Focus J: *Microtus brandti* plague focus of the Xilin gol League Plateau (MBPF). Focus K: *Apodemus chevrieri–Eothenomys miletus* plague focus of the Highlands of western Yunnan Province (ACEMPF). Focus L: *Rattus flavipectus plague* focus of the Yunnan‐Fujian‐Guangdong provinces (RFPF).

The STRUCTURE analysis splited the *Y. pestis* into eight genetic groups (Figure 4a) as the highest Δ*K* value was observed at *K* = 8 (Figure 4b). High admixture between clusters was observed in Group 1 (Figure 4b, colored in red). China strains were found predominantly in Group 6 (28.4%), but without Groups 7 and 8. There were four branching populations (2.MED0, 2.MED1, 2.MED2, 2.MED3) in Group 2; two (2.ANT1 and 2.ANT2) in Group 3; six (0.ANT2, 0.ANT3, 1.IN4, 3.ANT1, 3.ANT2 and 4.ANT1) in Group 4; twelve (1.ANT, 1.ANT1, 1.IN1, 1.IN2, 1.IN3, 1.IN4, 1.ORI1, 1.ORI2, 1.ORI3, 1.ORI4, 1.ORI5, and 1.ORI6) in Group 5; five (0.ANT1, 0.PE4A, 0.PE4B, 0.PE4C and 0.PE7) in Group 6; one (0.ANT1) in Group 7; and one (0.PE2) in Group 8 (Figure 4a).

Population structure analysis of the Chinese *Y. pestis* showed that different natural foci cluster as distinct genetic groups except three foci: MHPF includes all five kinds of genetic groups (Groups 2, 3, 4, 5, 6) and *A. chevrieri–E. miletus* plague focus of the Highlands of western Yunnan Province includes two kinds of genetic groups (4, 5); the MBSUPF includes two genetic groups with Group 6 being predominant (94.5%). Each of the other foci has its own distinct group (Figure 4a). The MHPF displayed relatively high genetic diversity, suggesting that it has remained separated in these regions.

### 3.5 Pan‐genome construction and SNP distance among different natural foci

**FIGURE 5:**
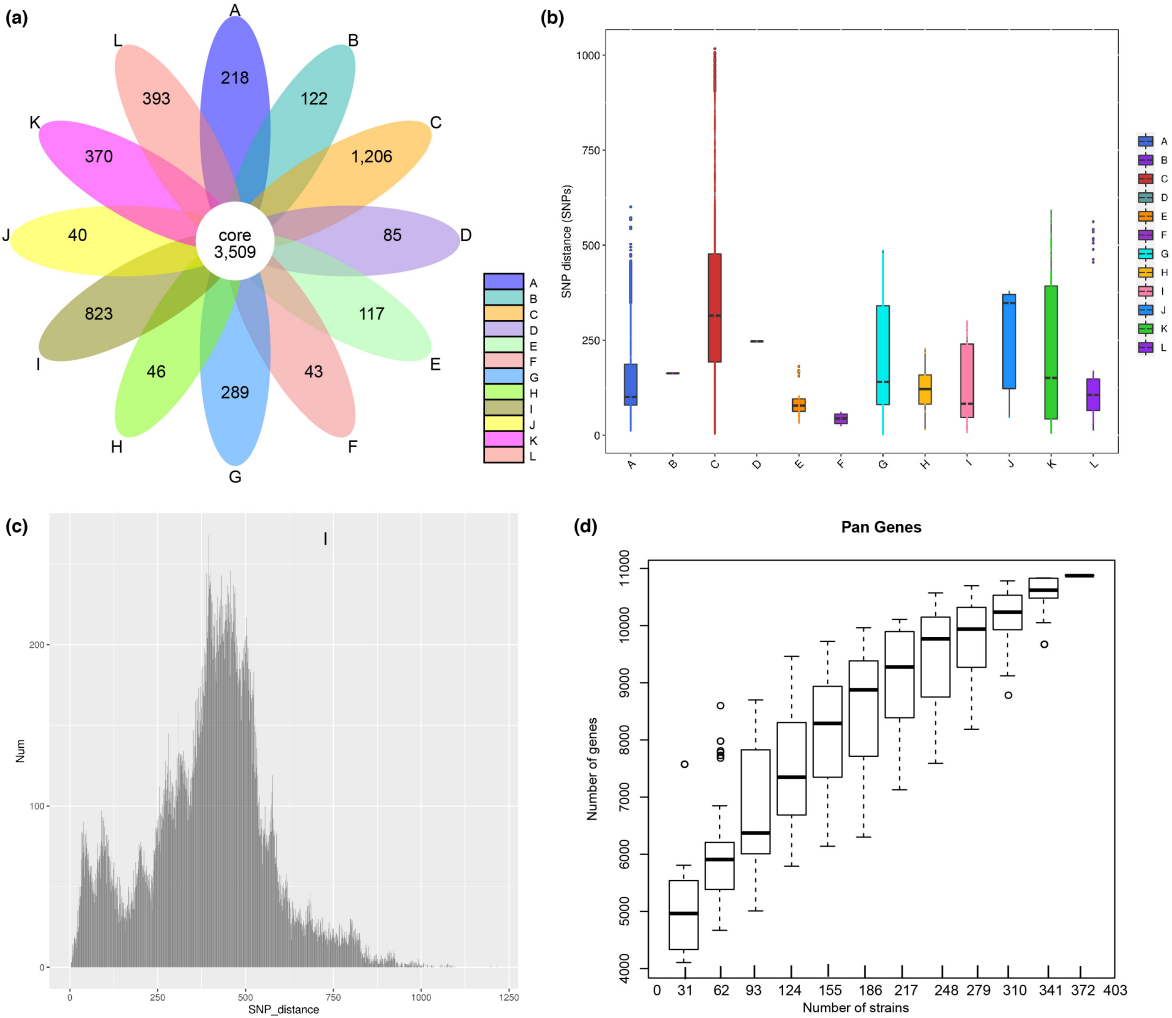
Pan‐genome analysis. (a) Distribution of core genes and accessory genes of different natural foci. Different colors denote different natural foci. (b) Box plot of pairwise SNP distances within groups of 12 natural foci. Box plots depict the upper, median, and lower quartiles of the ratios; individual dots indicate outliers that lie outside of 1.5 times the interquartile range. (c) Histograms of pairwise SNP distance distribution in 626 isolates. (d) Correlations between the number of the pan genes and the number of *Yersinia pestis* genomic sequences. Focus A: *Marmota baibacina–Spermophilus undulats* plague focus of the Tianshan Mountains. Focus B: *Marmota caudata* plague focus of the Pamir Plateau. Focus C: *Marmota himalayana* plague focus of the Qinghai–Tibet Plateau. Focus D: *Marmota sibirica* plague focus of the Hulun Buir Plateau. Focus E: *Spermophilus dauricus* plague focus of the Songliao Plain. Focus F: *Spermoophilus dauricus alaschanicus* plague focus of the Loess Plateau of Gansu and Ningxia provinces. Focus G: *Meriones unguiculatus* plague focus of the Inner Mongolian Plateau. Focus H: *Rhombomys opimus* plague focus of the Junggar Basin. Focus I: *Microtus foscus* plague natural focus of the Qinghai–Tibet Plateau. Focus J: *Microtus brandti* plague focus of the Xilin gol League Plateau.Focus K: *Apodemus chevrieri–Eothenomys miletus* plague focus of the Highlands of western Yunnan Province. Focus L: *Rattus flavipectus plague* focus of the Yunnan‐Fujian‐Guangdong provinces.

Analysis of this combined set of 626 isolates revealed that 3509 core genes were detected (Figure 5a). The pairwise SNP distance among strains isolated from different plague foci were quite different, the clades of the MHPF had larger SNP distances than other foci (Figure 5b). The SNP distance within the same plague foci was different (Figure 4b, Figure S2). There are two peaks in the pairwise SNP distance distribution, one below 100 and one below 400 SNPs (Figure 5c). The number of the pan genes increased together with the number of *Y. pestis* genomic sequences (Figure 5d).

The number of core genes decreased with the increase of strains (Figure S1). The distribution of unique genes in each natural focus varied widely, varying from 40 to 1206, suggesting a high degree of genetic diversity among the foci. The MHPF had the highest number of unique genes (1206 genes), and the MBPF had the lowest (40 genes) (Figure 5a), which also demonstrated that the former has a higher diversity than the latter. The MHPF genome was heavily enriched in genes related to mobilome, prophages, and transposons; but MBPF genome was heavily enriched in genes related to carbohydrate transport and metabolism. Also, we cannot ignored the different sampling number among each focus effect of the unique genes in the pan‐genome.

**FIGURE 6:**
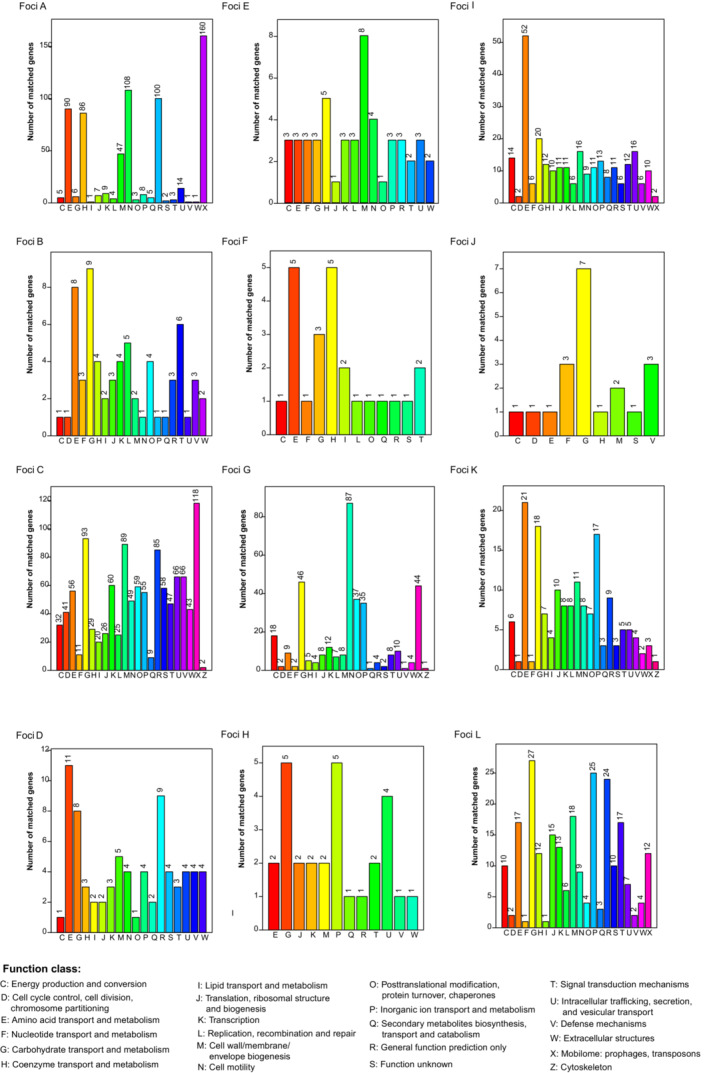
Distribution of COG categories in the unique genomes of different natural foci. Numbers of genes assigned to COG categories are shown. Focus A: *Marmota baibacina–Spermophilus undulats* plague focus of the Tianshan Mountains. Focus B: *Marmota caudata* plague focus of the Pamir Plateau. Focus C: *Marmota himalayana* plague focus of the Qinghai–Tibet Plateau. Focus D: *Marmota sibirica* plague focus of the Hulun Buir Plateau. Focus E: *Spermophilus dauricus* plague focus of the Songliao Plain. Focus F: *Spermoophilus dauricus alaschanicus* plague focus of the Loess Plateau of Gansu and Ningxia provinces. Focus G: *Meriones unguiculatus* plague focus of the Inner Mongolian Plateau. Focus H: *Rhombomys opimus* plague focus of the Junggar Basin. Focus I: *Microtus foscus* plague natural focus of the Qinghai–Tibet Plateau. Focus J: *Microtus brandti* plague focus of the Xilin gol League Plateau. Focus K: *Apodemus chevrieri–Eothenomys miletus* plague focus of the Highlands of western Yunnan Province. Focus L: *Rattus flavipectus* plague focus of the Yunnan‐Fujian‐Guangdong provinces.

The distribution of COG categories in the unique genomes of different natural foci varied. The unique genes of four out of 12 foci were mainly enriched in amino acid transport and metabolism, including MFPF, SDAPF, ACEMPF, and MSPF. The unique genes of MCPF, RFPF, and MBPF were all mainly enriched in functions of carbohydrate transport and metabolism. The unique genes of MHPF and MBSUPF were all mainly enriched in functions of the mobilome, prophages, and transposons (Figure 6), indicating that phages and prophages have played a significant role in the evolution and adaptation of the MHPF in different environments (Figure 6). However the enrichments observed for certain foci may be due to a specific lineage within the foci.

## 4 DISCUSSION

### 4.1 The influence of the Qinghai–Tibet plateau in the evolution of *Yersinia pestis*

Plague is a typical natural focus infection disease. It is currently endemic in restricted areas in the world (Dubyanskiy & Yeszhanov, 2016). The bacterial genome has changed over the course of a long evolutionary history, adapting through interactions with hosts and the local environment (Papadopoulos et al., 1999). This dynamic processes have led to genetic variation in *Y*. *pestis*, and it is important to explore this variation and its outcomes to be able to control and/or prevent further plague outbreaks.

In our study, more than 20 kinds of hosts and vectors from different parts of the world were analyzed. *Marmota* is currently considered the most important reservoir causing human outbreaks and maintaining plague persistence. The Qinghai–Tibet plateau has unique geographic features, and is the highest‐altitude plague host area in the world. It is the biggest and one of the most active areas of animal and human plague in China with a large variety of plague animals and strong virulence of plague bacteria (Ge et al., 2015; He et al., 2021; Wang et al., 2011). Moreover, in addition to the main host (marmot), the secondary hosts have appeared from time to time in recent years (Dai et al., 2018, 2019; Ge et al., 2015). The diversifying natural selection possibly coincides with the complex spatial epidemiology and genetic diversity of *Y. pestis* in Qinghai–Tibet. The remaining 11 different foci have their major genotypes of *Y. pestis*, and each genotype is confined to one or few vectors and reservoirs (Figure 2a). The diversity of hosts in each of the focus showed MHPF had the most diversity of host (Figure 2b). This suggests that the ecological conditions, geography, vectors, and reservoir species are key factors influencing the spatial and temporal evolution of the *Y. pestis* genome.

### 4.2 Geological barriers play an important role in the maintenance of local *Yersinia pestis* species and block the introduction of non‐native strains

**FIGURE 7:**
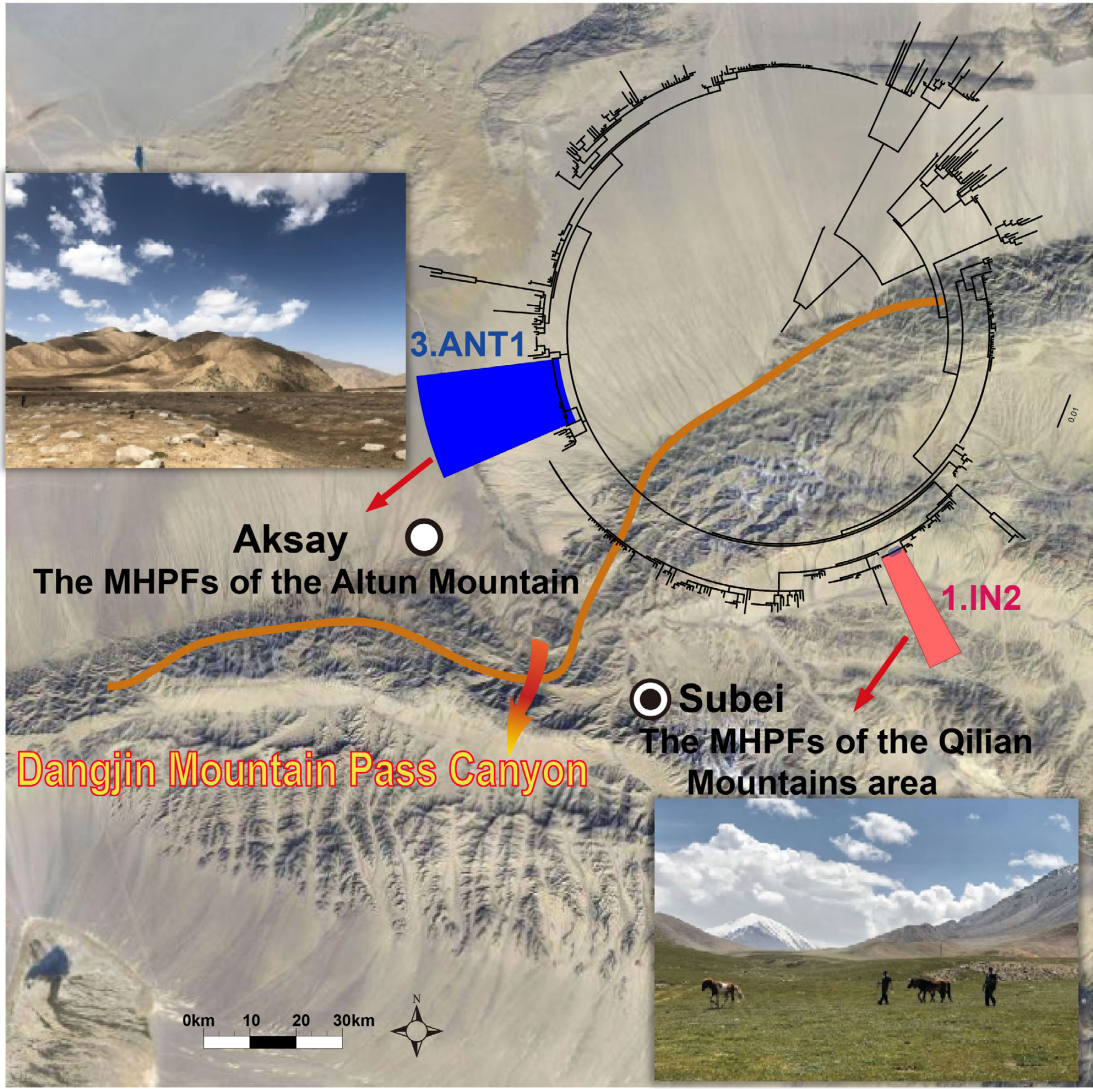
The different natural landscape of the Altun Mountain (Aksay) and the Qilian Mountains (Subei) in Gansu province. The two area are divided into two parts by the Dangjin Mountain Pass Canyon, and the natural landscape are obviously different. The former is dominated by desert and semi‐desert grasslands with 3.ANT1 genotype of *Yersinia pestis*, while the latter is dominated by alpine grassland meadows with 1.IN2 genotype.

The host and vector population dynamics and human movement may increase the chances of exposure and infection to drive the genome evolution (Cui et al., 2020; He et al., 2021). Geological barriers have strong impacts on the maintenance of local *Y. pestis* species and block the introduction of non‐native strains. The MHPFs of the Altun Mountain (Aksay) and Qilian Mountains (Subei) in Gansu province are basically have the same hosts and vectors, and they are geographically adjacent to each other. However, the area is divided into two parts by the Dangjin Mountain Pass Canyon, and the natural landscape is obviously different. The former is dominated by desert and semi‐desert grasslands, while the latter is dominated by alpine grassland meadows (Figure 7). The genotypes of *Y. pestis* in these two foci are obviously different due to the geographic barriers and other factors such as temperature, rainfall, biome, geography, and anthropogenic impact. Geography shaped the genetic diversity of *Y. pestis* through the reintroduction of new strains of hosts and/or the pathogen between different areas which may play an important role for the persistence of *Y. pestis*. The unique genes of MHPF enriched in mobilome functions (prophages, transposons), and this suggested that *Y. pestis* varied with survival selective pressure to balance the natural selection through horizontal gene transfer (HGT) during its expansion and adaptation to new niches.

### 4.3 Different hosts, vectors, and specific ecological landscape shaping various niches for *Yersinia pestis*

An interactive complex (ecological niche) formed by hosts, fleas, and *Y. pestis* affect the maintenance and evolution of *Y. pestis*. Temperature and humidity influence the development and survival of the two main flea vectors. Although some foci are geographically close, the genotypes of these foci varied with the main host species. Both the MHPF and MFPF are located on the Qinghai–Tibet Plateau with almost the same biotoype, but the genomes are highly different. There are two different kinds of plague foci in Yunnan province: *A. chevrieri–E. miletus* plague focus of the Highlands of western Yunnan Province (mainly with 1.IN3 and 1.IN4), and the *R. flavipectus* plague focus of the Yunnan‐Fujian‐Guangdong provinces (mainly with 1.ORI2). The former is the wild rodents host and latter is house mouse host. It is reported that the third pandemic was caused by 1.ORI group that evolved approximately 200 years ago (Morelli et al., 2010). According to our study results, the wild rodent isolates which belongs to 1.IN3/4 subpopulation might have evolved from MHPF and then spread to and established natural plague foci in the mouse host (the *R. flavipectus* plague focus of the Yunnan‐Fujian‐Guangdong provinces). It was previously concluded that the oriental and medieval spread of plague originated from the ancient typical *Y. pestis* (Morelli et al., 2010).

At the same time, in some distant foci with similar main host species, the *Y. pestis* are quite homogeneous. For example, among the plague focus of *Spermophilus*, MBSUPF is located in northwestern China, 94.5% of which are 0.ANT1 type; while *S. dauricus* plague focus of the Songliao Plain is located in northeastern China, and 90.9% of which are 4.ANT3 type. Although the two foci are thousands of kilometers apart, and involve two different *Spermophilus* species, the genomes belong to a large evolutionary branch. The MFPF is located on the Qinghai–Tibet Plateau, far from the MBPF, which is located on the Inner Mongolia Plateau, but the strains isolated from them are almost identical in pathogenicity and biochemical and biological characteristics: both cause only limited animal plague and are not pathogenic to humans (Song et al., 2004). They both belong to the same genotype (0.PE4C), 0.PE4 is ancestral to the polytomy that gave rise to 3.ANT1 of MHPF. The same is true with 2.MED1 of the *R. opimus* plague focus in the Junggar Basin and 2.MED3 of the *M. unguiculatus* plague focus on the Inner Mongolian Plateau.

The genome of *Y. pestis* in China is variable in different geographic environments and host populations among different natural foci. *Y. pestis* can adapt to different kinds of hosts, vectors, and specific ecological landscape, until a stable genotype is formed in each geographic region. Geological barriers have strong impacts on the maintenance of local *Y. pestis* species and block the introduction of non‐native strains. *Y. pestis* evolved in MHPF showed the most complex genomic diversity. The strains from MHPF is ancestral to the polytomy of branches 1–4, that gave rise to other populations via multiple independent radiations. Our study will definitely promote the application and research of space epidemiology and genomic epidemiology in plague control.

## AUTHOR CONTRIBUTIONS

**Junrong Liang:** Formal analysis (equal); methodology (equal); writing – original draft (equal); writing – review and editing (equal). **Ran Duan:** Data curation (equal); investigation (equal); writing – original draft (equal). **Shuai Qin:** Data curation (equal); formal analysis (equal); writing – original draft (equal). **Dongyue Lv:** Data curation (equal); investigation (equal); methodology (equal); visualization (equal). **Zhaokai He:** Investigation (equal); methodology (equal); resources (equal). **Haoran Zhang:** Investigation (equal); resources (equal). **Qun Duan:** Data curation (equal); software (equal). **Jinxiao Xi:** Data curation (equal); supervision (equal). **Hua Chun:** Methodology (equal); software (equal); visualization (equal). **Guoming Fu:** Investigation (equal); visualization (equal). **Xiaojin Zheng:** Data curation (equal); resources (equal). **Deming Tang:** Methodology (equal); software (equal). **Weiwei Wu:** Formal analysis (equal); investigation (equal); visualization (equal). **Haonan Han:** Data curation (equal); formal analysis (equal); validation (equal). **Huaiqi Jing:** Conceptualization (equal); funding acquisition (equal); supervision (equal). **Xin Wang:** Conceptualization (equal); funding acquisition (equal); methodology (equal); visualization (equal).

## ACKNOWLEDGEMENTS

The authors thank Charlesworth author services for their critical editing and helpful comments regarding our article.

## FUNDING INFORMATION

This work was supported by the National Key Research and Development Program of China 2022YFC2602203 and National Sci‐Tech Key Project (grant nos. 2018ZX10713‐003‐002 and 2018ZX10713‐001‐002).

## CONFLICT OF INTEREST STATEMENT

The authors declare no conflicts of interest.

## Supporting information


Figure S1.

Figure S2.

Figure S3.

Table S1.

Table S2.

## Data Availability

Genetic data: Raw sequence reads are deposited in the SRA (BioProject 811346).

## References

[ece310387-bib-0001] Achtman, M. , Morelli, G. , Zhu, P. , Wirth, T. , Diehl, I. , Kusecek, B. , Vogler, A. J. , Wagner, D. M. , Allender, C. J. , Easterday, W. R. , Chenal‐Francisque, V. , Worsham, P. , Thomson, N. R. , Parkhill, J. , Lindler, L. E. , Carniel, E. , & Keim, P. (2004). Microevolution and history of the plague bacillus, *Yersinia pestis* . Proceedings of the National Academy of Sciences of the United States of America, 101, 17837–17842. 15598742 10.1073/pnas.0408026101 PMC535704

[ece310387-bib-0002] Achtman, M. , Zurth, K. , Morelli, G. , Torrea, G. , Guiyoule, A. , & Carniel, E. (1999). *Yersinia pestis*, the cause of plague, is a recently emerged clone of *Yersinia pseudotuberculosis* . Proceedings of the National Academy of Sciences of the United States of America, 96, 14043–14048. 10570195 10.1073/pnas.96.24.14043 PMC24187

[ece310387-bib-0003] Ben‐Ari, T. , Neerinckx, S. , Agier, L. , Cazelles, B. , Xu, L. , Zhang, Z. , Fang, X. , Wang, S. , Liu, Q. , & Stenseth, N. C. (2012). Identification of Chinese plague foci from long‐term epidemiological data. Proceedings of the National Academy of Sciences of the United States of America, 109, 8196–8201. 22570501 10.1073/pnas.1110585109 PMC3361404

[ece310387-bib-0004] Bos, K. I. , Herbig, A. , Sahl, J. , Waglechner, N. , Fourment, M. , Forrest, S. A. , Klunk, J. , Schuenemann, V. J. , Poinar, D. , Kuch, M. , Golding, G. B. , Dutour, O. , Keim, P. , Wagner, D. M. , Holmes, E. C. , Krause, J. , & Poinar, H. N. (2016). Eighteenth century *Yersinia pestis* genomes reveal the long‐term persistence of an historical plague focus. eLife, 5, e12994. 10.7554/eLife.12994 PMC4798955 26795402

[ece310387-bib-0005] Bush, S. J. (2021). Generalizable characteristics of false‐positive bacterial variant calls. Microbial Genomics, 7(8), 000615. 34346861 10.1099/mgen.0.000615 PMC8549357

[ece310387-bib-0006] Croucher, N. J. , Page, A. J. , Connor, T. R. , Delaney, A. J. , Keane, J. A. , Bentley, S. D. , Parkhill, J. , & Harris, S. R. (2015). Rapid phylogenetic analysis of large samples of recombinant bacterial whole genome sequences using Gubbins. Nucleic Acids Research, 43, e15. 25414349 10.1093/nar/gku1196 PMC4330336

[ece310387-bib-0007] Cui, Y. , Schmid, B. V. , Cao, H. , Dai, X. , Du, Z. , Ryan Easterday, W. , Fang, H. , Guo, C. , Huang, S. , Liu, W. , & Qi, Z. (2020). Evolutionary selection of biofilm‐mediated extended phenotypes in *Yersinia pestis* in response to a fluctuating environment. Nature Communications, 11, 281. 10.1038/s41467-019-14099-w PMC6962365 31941912

[ece310387-bib-0008] Cui, Y. , Yu, C. , Yan, Y. , Li, D. , Li, Y. , Jombart, T. , Weinert, L. A. , Wang, Z. , Guo, Z. , Xu, L. , Zhang, Y. , Zheng, H. , Qin, N. , Xiao, X. , Wu, M. , Wang, X. , Zhou, D. , Qi, Z. , Du, Z. , … Yang, R. (2013). Historical variations in mutation rate in an epidemic pathogen, *Yersinia pestis* . Proceedings of the National Academy of Sciences of the United States of America, 110, 577–582. 23271803 10.1073/pnas.1205750110 PMC3545753

[ece310387-bib-0009] Dai, R. , Qi, M. , Xiong, H. , Yang, X. , He, J. , Zhang, Z. , Yang, H. , Jin, J. , Li, X. , Xin, Y. , Yang, Y. , Li, C. , Li, Z. , Xu, J. , Wang, Z. , Li, W. , & Wei, B. (2019). Serological epidemiological investigation of Tibetan sheep (*Ovis aries*) plague in Qinghai, China. Vector Borne and Zoonotic Diseases, 19, 3–7. 30256745 10.1089/vbz.2017.2257

[ece310387-bib-0010] Dai, R. , Wei, B. , Xiong, H. , Yang, X. , Peng, Y. , He, J. , Jin, J. , Wang, Y. , Zha, X. , Zhang, Z. , Liang, Y. , Zhang, Q. , Xu, J. , Wang, Z. , & Li, W. (2018). Human plague associated with Tibetan sheep originates in marmots. PLoS Neglected Tropical Diseases, 12, e0006635. 30114220 10.1371/journal.pntd.0006635 PMC6095483

[ece310387-bib-0011] de Sena, B. G. , & Smith, A. D. (2019). Falco: High‐speed FastQC emulation for quality control of sequencing data. F1000Research, 8, 1874. 33552473 10.12688/f1000research.21142.1 PMC7845152

[ece310387-bib-0012] Dubyanskiy, V. M. , & Yeszhanov, A. B. (2016). Ecology of *Yersinia pestis* and the epidemiology of plague. Advances in Experimental Medicine and Biology, 918, 101–170. 27722862 10.1007/978-94-024-0890-4_5

[ece310387-bib-0013] Evanno, G. , Regnaut, S. , & Goudet, J. (2005). Detecting the number of clusters of individuals using the software STRUCTURE: A simulation study. Molecular Ecology, 14, 2611–2620. 15969739 10.1111/j.1365-294X.2005.02553.x

[ece310387-bib-0014] Fang, X. Y. , Xu, L. , Liu, Q. Y. , & Zhang, R. Z. (2011). Eco‐geographic landscapes of natural plague foci in China I. Eco‐geographic landscapes of natural plague foci. Zhonghua Liu Xing Bing Xue Za Zhi, 32, 1232–1236. 22336607

[ece310387-bib-0015] Fu, L. , Niu, B. , Zhu, Z. , Wu, S. , & Li, W. (2012). CD‐HIT: Accelerated for clustering the next‐generation sequencing data. Bioinformatics, 28, 3150–3152. 23060610 10.1093/bioinformatics/bts565 PMC3516142

[ece310387-bib-0016] Ge, P. , Xi, J. , Ding, J. , Jin, F. , Zhang, H. , Guo, L. , Zhang, J. , Li, J. , Gan, Z. , Wu, B. , Liang, J. , Wang, X. , & Wang, X. (2015). Primary case of human pneumonic plague occurring in a Himalayan marmot natural focus area Gansu Province, China. International Journal of Infectious Diseases, 33, 67–70. 25555623 10.1016/j.ijid.2014.12.044

[ece310387-bib-0017] He, Z. , Wei, B. , Zhang, Y. , Liu, J. , Xi, J. , Ciren, D. , Qi, T. , Liang, J. , Duan, R. , Qin, S. , Lv, D. , Chen, Y. , Xiao, M. , Fan, R. , Song, Z. , Jing, H. , & Wang, X. (2021). Distribution and characteristics of human plague cases and *Yersinia pestis* isolates from 4 Marmota plague foci, China, 1950–2019. Emerging Infectious Diseases, 27, 2544–2553. 34545784 10.3201/eid2710.202239 PMC8462326

[ece310387-bib-0018] Ito, K. , & Murphy, D. (2013). Application of ggplot2 to pharmacometric graphics. CPT: Pharmacometrics & Systems Pharmacology, 2, e79. 24132163 10.1038/psp.2013.56 PMC3817376

[ece310387-bib-0019] Khan, I. A. (2004). Plague: The dreadful visitation occupying the human mind for centuries. Transactions of the Royal Society of Tropical Medicine and Hygiene, 98, 270–277. 15109549 10.1016/S0035-9203(03)00059-2

[ece310387-bib-0020] Langmead, B. , & Salzberg, S. L. (2012). Fast gapped‐read alignment with bowtie 2. Nature Methods, 9, 357–359. 22388286 10.1038/nmeth.1923 PMC3322381

[ece310387-bib-0021] Letunic, I. , & Bork, P. (2021). Interactive tree of life (iTOL) v5: An online tool for phylogenetic tree display and annotation. Nucleic Acids Research, 49, W293–W296. 33885785 10.1093/nar/gkab301 PMC8265157

[ece310387-bib-0022] Lynteris, C. (2017). A ‘suitable soil’: Plague's urban breeding grounds at the dawn of the third pandemic. Medical History, 61, 343–357. 28604289 10.1017/mdh.2017.32 PMC5471971

[ece310387-bib-0023] Meerburg, B. G. , Singleton, G. R. , & Kijlstra, A. (2009). Rodent‐borne diseases and their risks for public health. Critical Reviews in Microbiology, 35, 221–270. 19548807 10.1080/10408410902989837

[ece310387-bib-0024] Miller, S. A. , Dykes, D. D. , & Polesky, H. F. (1988). A simple salting out procedure for extracting DNA from human nucleated cells. Nucleic Acids Research, 16, 1215. 3344216 10.1093/nar/16.3.1215 PMC334765

[ece310387-bib-0025] Morelli, G. , Song, Y. , Mazzoni, C. J. , Eppinger, M. , Roumagnac, P. , Wagner, D. M. , Feldkamp, M. , Kusecek, B. , Vogler, A. J. , Li, Y. , Cui, Y. , Thomson, N. R. , Jombart, T. , Leblois, R. , Lichtner, P. , Rahalison, L. , Petersen, J. M. , Balloux, F. , Keim, P. , … Achtman, M. (2010). *Yersinia pestis* genome sequencing identifies patterns of global phylogenetic diversity. Nature Genetics, 42, 1140–1143. 21037571 10.1038/ng.705 PMC2999892

[ece310387-bib-0026] Papadopoulos, D. , Schneider, D. , Meier‐Eiss, J. , Arber, W. , Lenski, R. E. , & Blot, M. (1999). Genomic evolution during a 10,000‐generation experiment with bacteria. Proceedings of the National Academy of Sciences of the United States of America, 96, 3807–3812. 10097119 10.1073/pnas.96.7.3807 PMC22376

[ece310387-bib-0027] Perry, R. D. , & Fetherston, J. D. (1997). *Yersinia pestis* – Etiologic agent of plague. Clinical Microbiology Reviews, 10, 35–66. 8993858 10.1128/cmr.10.1.35 PMC172914

[ece310387-bib-0028] Prjibelski, A. , Antipov, D. , Meleshko, D. , Lapidus, A. , & Korobeynikov, A. (2020). Using SPAdes de novo assembler. Current Protocols in Bioinformatics, 70, e102. 32559359 10.1002/cpbi.102

[ece310387-bib-0029] Rabiee, M. H. , Mahmoudi, A. , Siahsarvie, R. , Krystufek, B. , & Mostafavi, E. (2018). Rodent‐borne diseases and their public health importance in Iran. PLoS Neglected Tropical Diseases, 12, e0006256. 29672510 10.1371/journal.pntd.0006256 PMC5908068

[ece310387-bib-0030] Seemann, T. (2014). Prokka: Rapid prokaryotic genome annotation. Bioinformatics, 30, 2068–2069. 24642063 10.1093/bioinformatics/btu153

[ece310387-bib-0031] Song, Y. , Tong, Z. , Wang, J. , Wang, L. , Guo, Z. , Han, Y. , Zhang, J. , Pei, D. , Zhou, D. , Qin, H. , Pang, X. , Han, Y. , Zhai, J. , Li, M. , Cui, B. , Qi, Z. , Jin, L. , Dai, R. , Chen, F. , … Yang, R. (2004). Complete genome sequence of *Yersinia pestis* strain 91001, an isolate avirulent to humans. DNA Research, 11, 179–197. 15368893 10.1093/dnares/11.3.179

[ece310387-bib-0032] Tatusov, R. L. , Galperin, M. Y. , Natale, D. A. , & Koonin, E. V. (2000). The COG database: A tool for genome‐scale analysis of protein functions and evolution. Nucleic Acids Research, 28, 33–36. 10592175 10.1093/nar/28.1.33 PMC102395

[ece310387-bib-0033] Wagner, G. P. , & Zhang, J. (2011). The pleiotropic structure of the genotype‐phenotype map: The evolvability of complex organisms. Nature Reviews. Genetics, 12, 204–213. 10.1038/nrg2949 21331091

[ece310387-bib-0034] Wang, H. , Cui, Y. , Wang, Z. , Wang, X. , Guo, Z. , Yan, Y. , Li, C. , Cui, B. , Xiao, X. , Yang, Y. , Qi, Z. , Wang, G. , Wei, B. , Yu, S. , He, D. , Chen, H. , Chen, G. , Song, Y. , & Yang, R. (2011). A dog‐associated primary pneumonic plague in Qinghai Province, China. Clinical Infectious Diseases, 52, 185–190. 21288842 10.1093/cid/ciq107

[ece310387-bib-0035] Wang, X. , Wei, X. , Song, Z. , Wang, M. , Xi, J. , Liang, J. , Liang, Y. , Duan, R. , Tian, K. , Zhao, Y. , Tang, G. , You, L. , Yang, G. , Liu, X. , Chen, Y. , Zeng, J. , Wu, S. , Luo, S. , Qin, G. , … Jing, H. (2017). Mechanism study on a plague outbreak driven by the construction of a large reservoir in Southwest China (surveillance from 2000–2015). PLoS Neglected Tropical Diseases, 11, e0005425. 28257423 10.1371/journal.pntd.0005425 PMC5352140

[ece310387-bib-0036] Zhou, D. , Han, Y. , Song, Y. , Tong, Z. , Wang, J. , Guo, Z. , Pei, D. , Pang, X. , Zhai, J. , Li, M. , Cui, B. , Qi, Z. , Jin, L. , Dai, R. , du, Z. , Bao, J. , Zhang, X. , Yu, J. , Wang, J. , … Yang, R. (2004). DNA microarray analysis of genome dynamics in *Yersinia pestis*: Insights into bacterial genome microevolution and niche adaptation. Journal of Bacteriology, 186, 5138–5146. 15262950 10.1128/JB.186.15.5138-5146.2004 PMC451624

[ece310387-bib-0037] Zietz, B. P. , & Dunkelberg, H. (2004). The history of the plague and the research on the causative agent *Yersinia pestis* . International Journal of Hygiene and Environmental Health, 207, 165–178. 15031959 10.1078/1438-4639-00259 PMC7128933

