# Supplementary figures and images for: The complex genomic diversity of Yersinia pestis on the long‐term plague foci in Qinghai–Tibet plateau
Source: Ecol Evol. 2023 Jul 28;13(8):e10387. doi: 10.1002/ece3.10387 (PMC10375460; doi:10.1002/ece3.10387)

## Core Genes

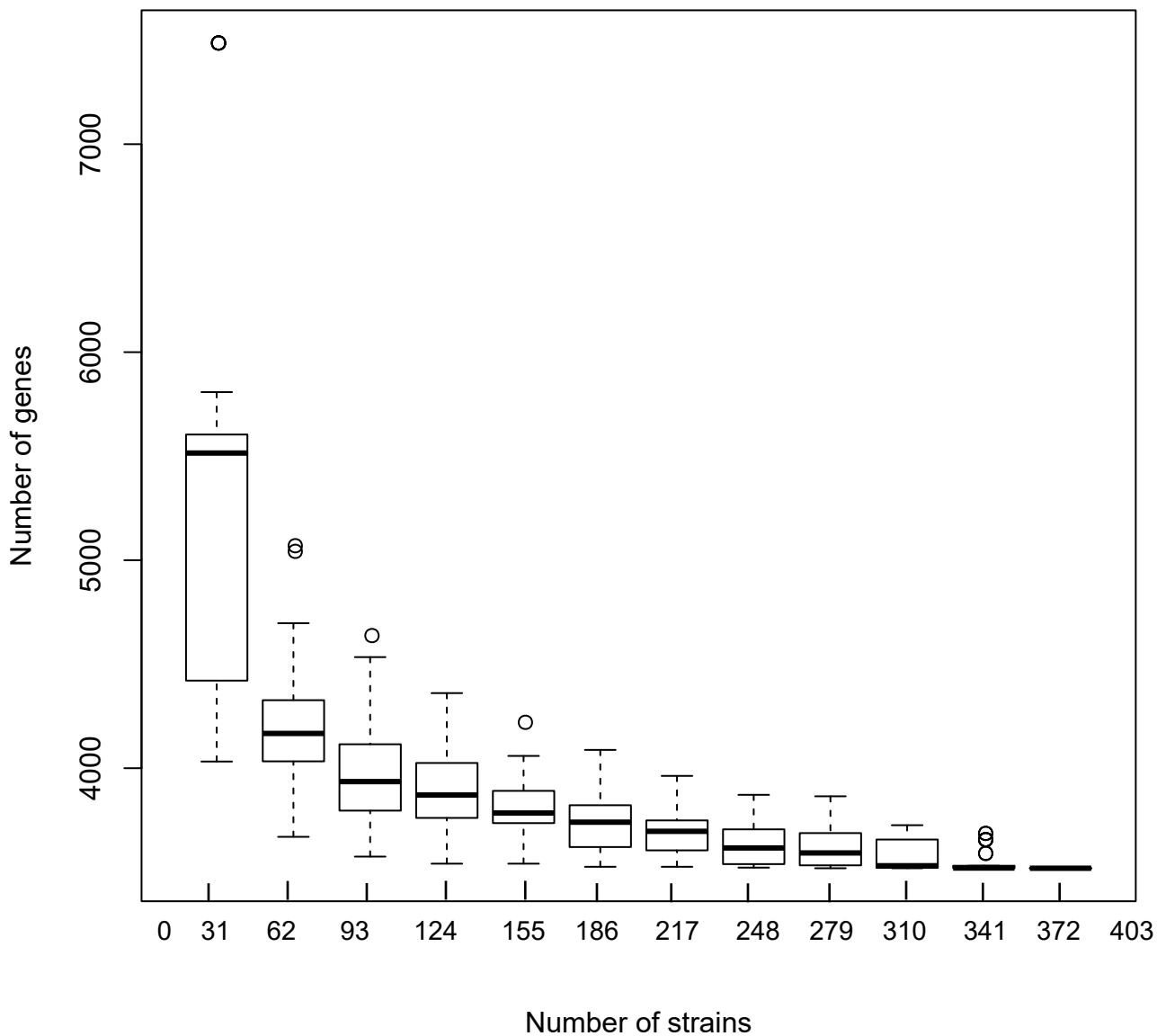

Supplement: Supplementary file 1 — Figure S1. [file ECE3-13-e10387-s002.pdf]

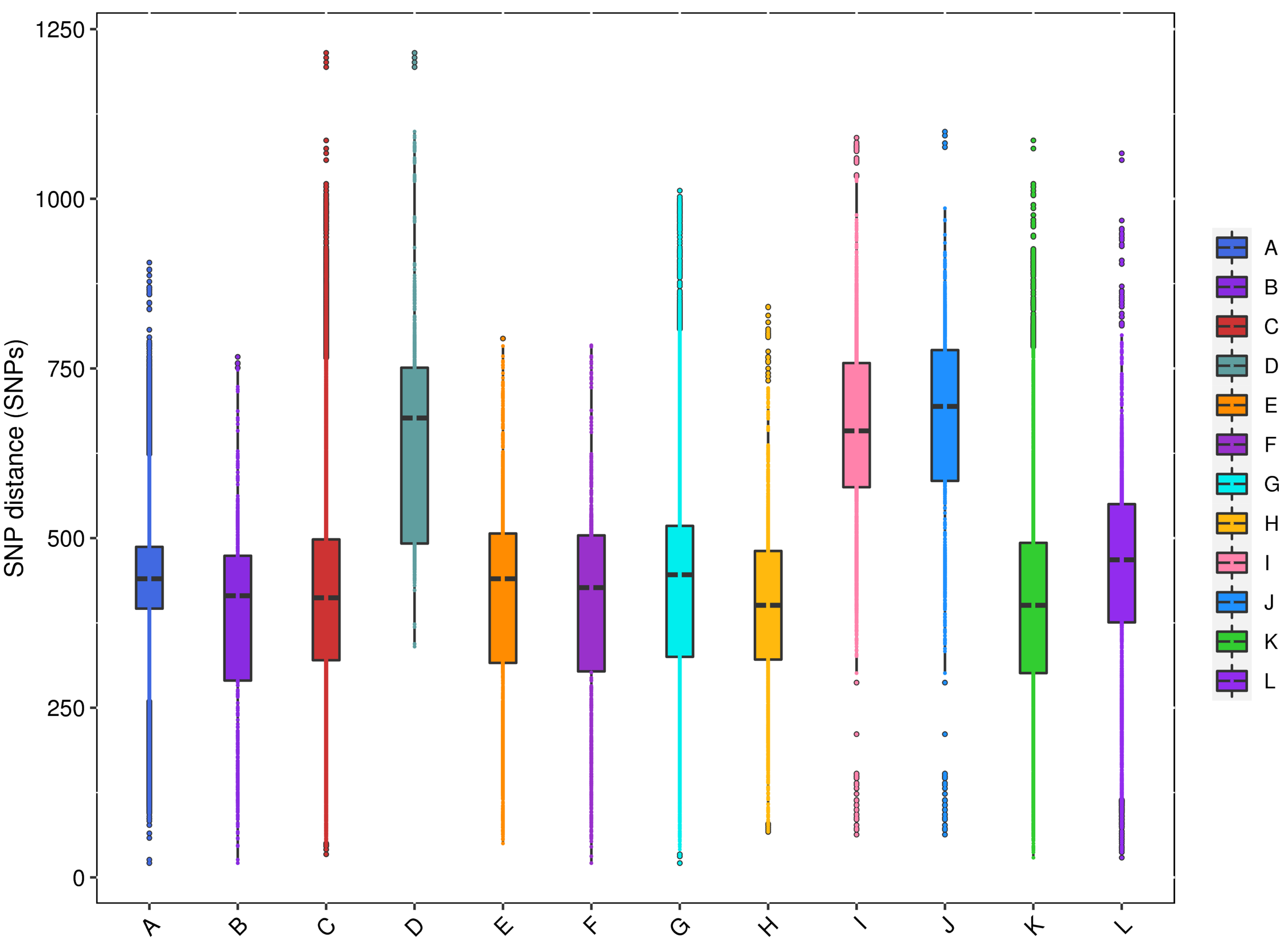

Supplement: Supplementary file 2 — Figure S2. [file ECE3-13-e10387-s003.pdf]
