# Supplementary material for: The complex genomic diversity of Yersinia pestis on the long‐term plague foci in Qinghai–Tibet plateau
Source: Ecol Evol. 2023 Jul 28;13(8):e10387. doi: 10.1002/ece3.10387 (PMC10375460; doi:10.1002/ece3.10387)
Supplement: Supplementary file 3 — Figure S3. [file ECE3-13-e10387-s005.pdf]

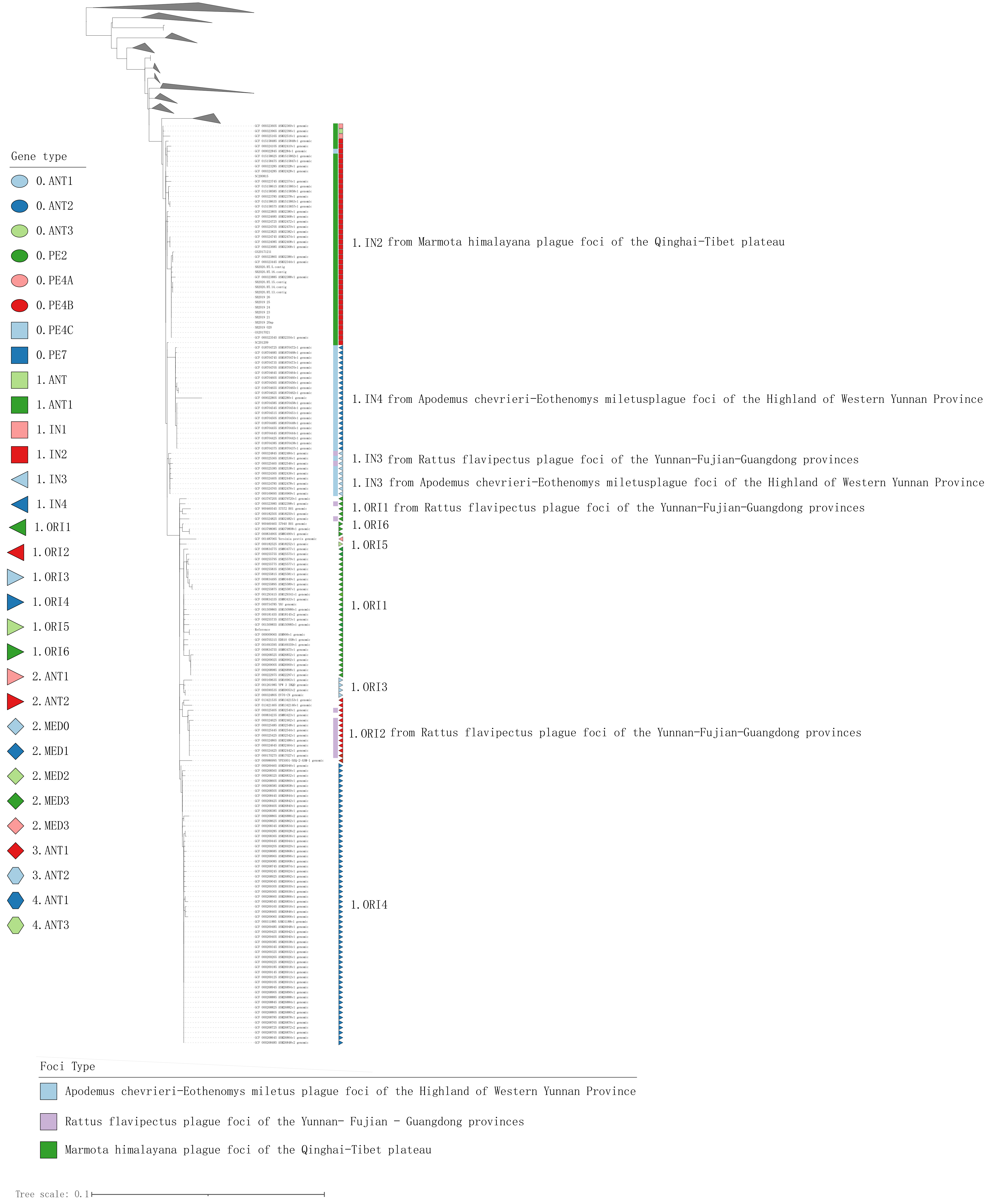
Gene type
0.ANT1
0.ANT2
0.ANT3
0.PE2
0.PE4A
0.PE4B
0.PE4C
0.PE7
1.ANT
1.ANT1
1.IN1
1.IN2
1.IN3
1.IN4
1.ORI1
1.ORI2
1.ORI3
1.ORI4
1.ORI5
1.ORI6
2.ANT1
2.ANT2
2.MED0
2.MED1
2.MED2
2.MED3
2.MED3
3.ANT1
3.ANT2
4.ANT1
4.ANT3
1.IN2 from Marmota himalayana plague foci of the Qinghai-Tibet plateau
1.IN4 from Apodemus chevrieri-Eothenomys miletusplague foci of the Highland of Western Yunnan Province
1.IN3 from Rattus flavipectus plague foci of the Yunnan-Fujian-Guangdong provinces
1.IN3 from Apodemus chevrieri-Eothenomys miletusplague foci of the Highland of Western Yunnan Province
1.ORI1 from Rattus flavipectus plague foci of the Yunnan-Fujian-Guangdong provinces
1.ORI6
1.ORI5
1.ORI1
1.ORI3
1.ORI2 from Rattus flavipectus plague foci of the Yunnan-Fujian-Guangdong provinces
1.ORI4
Foci Type
Apodemus chevrieri-Eothenomys miletus plague foci of the Highland of Western Yunnan Province
Rattus flavipectus plague foci of the Yunnan- Fujian - Guangdong provinces
Marmota himalayana plague foci of the Qinghai-Tibet plateau
Tree scale: 0.1
